# Patterns of enteric infections in a population-wide cohort study of sequelae, British Columbia, Canada

**DOI:** 10.1017/S0950268822001911

**Published:** 2022-12-14

**Authors:** Mahmood R. Gohari, Marsha Taylor, Melissa C. MacKinnon, Dimitra Panagiotoglou, Eleni Galanis, Gilaad G. Kaplan, Richard J. Cook, David M. Patrick, Steen Ethelberg, Shannon E. Majowicz

**Affiliations:** 1 School of Public Health Sciences, University of Waterloo, Waterloo, Canada; 2 British Columbia Centre for Disease Control, Vancouver, Canada; 3 Department of Epidemiology, Biostatistics and Occupational Health, McGill University, Montreal, Canada; 4 School of Population and Public Health, University of British Columbia, Vancouver, Canada; 5 Department of Medicine and Community Health Sciences, University of Calgary, Calgary, Canada; 6 Department of Statistics and Actuarial Science, University of Waterloo, Waterloo, Canada; 7 Department of Infectious Disease Epidemiology and Prevention, Statens Serum Institut, Copenhagen, Denmark; 8 Global Health Section, Department of Public Health, University of Copenhagen, Copenhagen, Denmark

**Keywords:** Burden of disease, enteric infection, foodborne infection, incidence rate, surveillance

## Abstract

We assessed patterns of enteric infections caused by 14 pathogens, in a longitudinal cohort study of sequelae in British Columbia (BC) Canada, 2005–2014. Our population cohort of 5.8 million individuals was followed for an average of 7.5 years/person; during this time, 40 523 individuals experienced 42 308 incident laboratory-confirmed, provincially reported enteric infections (96.4 incident infections per 100 000 person-years). Most individuals (38 882/40 523; 96%) had only one, but 4% had multiple concurrent infections or more than one infection across the study. Among individuals with more than one infection, the pathogens and combinations occurring most frequently per individual matched the pathogens occurring most frequently in the BC population. An additional 298 557 new fee-for-service physician visits and hospitalisations for enteric infections, that did not coincide with a reported enteric infection, also occurred, and some may be potentially unreported enteric infections. Our findings demonstrate that sequelae risk analyses should explore the possible impacts of multiple infections, and that estimating risk for individuals who may have had a potentially unreported enteric infection is warranted.

## Introduction

Enteric infections commonly transmitted by food are an important public health issue globally and in Canada [1, 2]. Beyond their acute phase (mainly self-limited vomiting and diarrhoea), sequelae such as haemolytic uremic syndrome, Guillain–Barré syndrome (GBS) and reactive arthritis can occur [3–6]. More than one pathogen can lead to a particular sequela (e.g. non-typhoidal salmonella and campylobacter can both lead to reactive arthritis), and more than one sequela is possible for some pathogens (e.g. campylobacter can lead to GBS and reactive arthritis) [4, 7]. Studies in the UK, Denmark, Sweden, the USA and Australia have used population-based longitudinal administrative health data to assess associations between enteric infections and various sequelae [3, 4, 7–16]. The advantage of this design is population-level coverage, however, several complexities surrounding the assessment of enteric infections in this context warrant consideration, as follows.

First, individuals may have multiple infections over the course of a longitudinal study with long follow-up, either with the same or different enteric pathogens. Certain studies address this by only including the first infection an individual has during the study [4, 8, 15, 16], although depending on the follow-up time for sequelae development post-infection, subsequent infections may still impact sequelae risk. Jess *et al*. managed this by censoring an individual's follow-up time at the date of their second infection [8]. Another issue is that individuals can have concurrent infections with more than one pathogen. Nielsen *et al*. managed this by excluding individuals concurrently infected with any other pathogens at the time of their campylobacter infection [16]. It is unclear from other population-based studies if or how individuals with multiple infections (either concurrently or over time) were managed when determining the risk of sequelae, nor how extensive these issues may be.

We conducted a retrospective, population-based cohort study of individuals in British Columbia (BC), Canada over 2005–2014, to estimate the long-term burden and risk of 15 sequelae associated with 14 infections commonly transmitted by food [17]. The objective of this research was to describe the epidemiology of new (i.e. incident) enteric infections caused by 14 pathogens commonly transmitted by food within the cohort, including the nature and extent of concurrent and recurrent infections, to help guide future sequelae risk estimation.

## Methods

### Study design and data

Our longitudinal cohort comprises all individuals registered in BC's provincial health insurance programme (Medical Services Plan (MSP)) at any time between 01 January 2005 to 31 December 2014. Enrolment in MSP is mandatory and the programme covers nearly all the BC population, with few exceptions such as members of the military [17, 18]. Full details of the study setting, MSP, methodology and administrative databases used and their linkage are detailed elsewhere [17]. Here, we descriptively analysed enteric infections occurring within the cohort, using information from three administrative databases linked at the individual level [19–21]: the individual's age, sex and unique study identifier; time spent in the study population (start date and number of days registered in MSP each study year); and the dates and International Classification of Disease (ICD) codes for all hospital admissions and all fee-for-service physician visits (excludes services provided via alternative plans including salaried, sessional and service agreement contracts; [22]) during the study period (2005–2014). Hereafter, we use ‘visit’ to mean fee-for-service physician visit, and ‘hospitalisation’ to mean hospital admission.

We defined individuals as having an enteric infection using BC's public health reportable disease database, specifically those with reports of laboratory-confirmed infections caused by one of 14 pathogens (the 13 listed in Table 1, plus *Clostridium botulinum*) with reported dates during our study period. For these reports, we had data on onset and reported dates, pathogen and the individual's unique study identifier that enabled linkage to the cohort. During 2005–2014, these infections were diagnosed predominantly by culture and microscopy (multiplex polymerase chain reaction (PCR) was not in use).

**Table 1.** Rates of incident enteric infections, by individuals' characteristics, in a longitudinal cohort in British Columbia, Canada (*n* = 5 819 344), 2005–2014

Characteristic	No. incident infections	Incidence rates per 100 000 person-years													
		Total	*Campylobacter*	Non-typhoidal *Salmonella*	*Giardia*	*Yersinia* (excluding *pestis*)	*Shigella*	Shiga toxin-producing *E. coli*	*Cryptosporidium*	Hepatitis A	*Cyclospora*	*Salmonella* Paratyphi	*Salmonella* Typhi	*Vibrio parahemolyticus*	*Listeria*
Sex															
Female	20 099	90.2	34.1	19.0	10.8	13.7	3.4	3.4	1.8	0.9	0.9	0.8	0.7	0.5	0.3
Male	22 209	102.3	40.4	19.2	16.6	11.3	5.0	2.8	2.1	1.0	0.9	0.9	0.8	1.0	0.4
Age (years)															
Median (range)	–	37.7 (0–101)	40.8 (0–100)	32.7 (0–98)	34.8 (1–101)	44.4 (0–100)	40.1 (1–92)	28.6 (0–101)	24.7 (1–90)	28.9 (1–90)	47.5 (1–88)	31.1 (0–91)	28.8 (1–84)	46.7 (14–93)	68.2 (0–98)
<1	465	108.8	27.2	55.1	6.8	10.7	1.9	1.4	2.7	0.5	0.0	1.0	0.2	0.0	1.1
1–4	2954	173.0	50.7	44.0	31.9	19.0	5.4	8.6	8.2	1.3	0.2	1.8	1.8	0.0	0.1
5–9	2069	94.5	25.5	24.2	19.5	9.1	3.4	4.7	4.2	1.9	0.2	0.8	0.9	0.0	0.0
10–14	1529	62.9	19.4	15.2	9.8	6.2	2.2	3.9	2.1	1.7	0.1	1.0	1.1	0.1	0.1
15–19	2171	79.2	32.0	18.6	7.8	8.1	2.3	4.8	2.5	1.3	0.4	0.8	0.4	0.2	0.1
20–24	3592	125.8	51.2	25.3	18.1	13.3	5.0	4.9	2.5	1.2	0.9	1.6	1.4	0.3	0.0
25–29	3697	128.8	49.6	25.9	19.1	15.0	6.0	3.0	3.4	1.3	1.1	1.4	1.6	1.0	0.2
30–39	5851	100.3	38.2	17.3	17.6	11.5	5.4	2.1	2.1	1.2	1.2	1.2	1.1	1.2	0.1
40–59	11 772	87.6	35.4	14.9	13.1	12.2	4.9	1.9	1.1	0.7	1.1	0.7	0.5	1.0	0.2
60+	8208	86.9	38.0	15.5	7.3	15.8	2.9	3.0	0.6	0.5	1.0	0.4	0.2	0.7	1.1

Because reportable disease data may not capture all infections [23], we also explored the presence of individuals who may have had an unreported enteric infection. To do this, we identified individuals with visits or hospitalisations for enteric infection. We defined ‘enteric infection’ as those with ICD code(s) for any of our 14 pathogens or for non-specific infectious gastroenteritis (Table 2). For visits and hospitalisations with any of these codes during the 10-year study, we compared the individual's visit/hospitalisation date to the onset date of their reported enteric infection (with any of the 14 pathogens), if applicable. For individuals with more than one reported infection, we compared the visit/hospitalisation date to the closest infection onset date (before or after). Visits/hospitalisations for enteric infections that did not coincide with a reported enteric infection were considered to represent potentially unreported enteric infections (e.g. sought care with an enteric infection but did not submit a stool sample).

**Table 2.** The number of fee-for-service physician visits and hospitalisations with International Classification of Diseases (ICD) codes for enteric infections and non-specific infectious gastroenteritis, that occurred in a longitudinal cohort in British Columbia, Canada (*n* = 5 819 344), 2005–2014

Pathogen	ICD-10 codes	ICD-9 codes	Total no. of visits with at least one code		Total no. of individuals with at least one code	
			Physician visits ^a^	Hospitalisations^b^	Physician visits^a^	Hospitalisations^b^
*Campylobacter*	A04.5	008.43	143	760	92	742
*Clostridium botulinum*	A05.1	005.1	172	13	46	12
*Cryptosporidium*	A07.2	007.4	34	45	25	39
*Cyclospora*	A07.4^c^	007.5	76	0	30	0
*Giardia*	A07.1	007.1	6193	137	2524	130
Hepatitis A	B15^c^, B15.0, B15.9	070.0, 070.1	16 059	212	3931	206
*Listeria*	A32^c^, A32.0, A32.1, A32.7, A32.8, A32.9	027.0	219	153	63	127
*Salmonella* spp., non-typhoidal	A02.0, A02.1, A02.2, A02.8, A02.9	003.0, 003.1, 003.2, 003.8, 003.9	28 897	903	6859	832
*Salmonella* Paratyphi and Typhi	A01^c^, A01.0, A01.1, A01.2, A01.3, A01.4	002.0, 002.1, 002.2, 002.3, 002.9	2899	264	1269	240
Shiga toxin-producing *E. coli*	A04.1, A04.3	008.02, 008.04	237	312	142	291
*Shigella*	A03.0, A03.1, A03.2, A03.3, A03.8, A03.9	004.0, 004.1, 004.2, 004.3, 004.8, 004.9	55 608	139	8837	128
*Vibrio parahaemolyticus*	A05.3	005.4	716	1–4^d^	315	1–4
*Yersinia* (excluding *pestis*)	A04.6	008.44	66	142	43	133
Non-specific infectious gastroenteritis	A04.9, A05.8, A05.9, A07.9, A08.4, A09, A09.0, K52.1	005.89, 005.9, 008.5, 007.9, 008.8, 009.0, 009.1, 009.2, 009.3, 558.2	433 114	57 935	182 333	52 623
One or more of the above codes			544 374	60 950	202 307	55 198

a The total no. physician visits for the whole study population across the study period was 1.1 billion, by 5 393 462 individuals.

b The total no. hospitalisations for the whole study population across the study period was 7.9 million, by 2 844 390 individuals.

c ICD code not in ICD-10-CA since 2006.

d Cell sizes between 1 and 4 are suppressed.

Because onset dates were missing for 98.8% (42 809/43 311) of the reported infections, we estimated missing onset dates by examining pathogen-specific median lag times between onset and reported dates in our data where available. Then, we compared calculated lags to lags for the same infections reported between 2015 and 2019, when a new database structure led to more consistent completion of the onset date field, and to those published for selected infections in BC [24]. Since these three sources had lag times that were either identical or very similar (maximum difference: 3 days; Supplementary Table S1; Supplementary Materials available on the Cambridge Core website), we used lags calculated from the 2015–2019 data (because they were based on the most cases and were available for all pathogens), and estimated missing onset dates by subtracting pathogen-specific median lags from reported dates.

The laboratory-confirmed reports in BC's public health database capture both incident infections (e.g. the first positive test result for new campylobacter infection), and any additional positive tests over the course of an existing infection (e.g. subsequent positive test results during said campylobacter infection). Thus, to identify incident infections, we determined pathogen-specific exclusionary periods, within which additional reports of that same pathogen were unlikely to represent a new infection. Because the duration of immunity following an enteric infection, other than hepatitis A, is unknown, we examined the time to subsequent report with the same pathogen within individuals. We interpreted results given current thinking that most bacterial infections confer immunity of several months' duration, while parasitic infections tend to last longer exhibiting prolonged clinical illness and related duration of immunity [25]. We defined exclusionary periods for all pathogens except hepatitis A, as it confers lifelong immunity and thus causes only one incident infection. We also applied an exclusionary period to define incident visits or hospitalisations for enteric infections. We considered additional visits or hospitalisations with any of the ICD codes (Table 2) within the exclusionary period unlikely to represent a new, distinct event. Finally, we identified infections with onset dates prior to the 01 January 2005 study start date and excluded them as non-incident.

Access to linked data for this study was facilitated by Population Data BC, under approvals from the BC Ministry of Health, the Data Stewardship Committee and the BC Centre for Disease Control together with the Panorama Data Governance Committee. Request for access to the exact data used here can be made by submitting a Data Access Request to Population Data BC (https://www.popdata.bc.ca; quote project 15-180, PIs: Majowicz and Galanis), and entering into research agreements with the data providers. Note that permission for Population Data BC to use our methods to re-create the cohort is granted by principal investigators Majowicz and Galanis. This study was approved by a University of Waterloo Research Ethics Committee (no 30645), the University of British Columbia Behavioral Research Ethics Board (no H16-00021) and McGill University's Institutional Review Board (no A03-M12- 19A).

### Analysis

Analyses were performed in SAS V.9.4 (SAS Institute, Cary, North Carolina, USA) and R (R Core Team, 2020; https://www.R-project.org/). We explored and reported details of any excluded or missing data. Per our data access agreement, cell counts <5 are reported as ‘1–4’ or are suppressed, though when such numbers are publicly available on the provincial disease surveillance website [26] we report them here with references. We summarised sex, month and pathogen, and determined the median and interquartile range (IQR) to summarise age. The Kruskal–Wallis test was used to determine significant differences in median ages and Fisher's exact test was used to determine significant differences in proportions [27]. Among individuals with >1 incident infection, we considered infections from different pathogens to occur at the same time during the 10-year study when onset dates occurred within 30 days.

All individuals in the cohort contributed time-at-risk for developing an incident infection. For individuals without infections, exact time-at-risk was calculated by tallying the total number of days registered in MSP during the study and by study year [27]. For individuals with incident infections, time-at-risk was calculated similarly, except time-at-risk stopped accruing at the onset date of the incident infection and resumed after the pathogen-specific exclusionary period. We calculated incidence rates by dividing the number of incident infections by the total time-at-risk in person-days [27]. We calculated overall and pathogen-specific incidence rates by year, sex and age category, and incidence rate ratios for sex and age category. We conducted two sensitivity analyses to examine the effect of excluding non-incident infections on our calculated incidence rates, and compared our incidence rates (calculated using incident reported infections and person-days at risk) to those published in annual provincial surveillance reports (calculated using all reported infections and the provincial population).

## Results

**Fig. 1.**
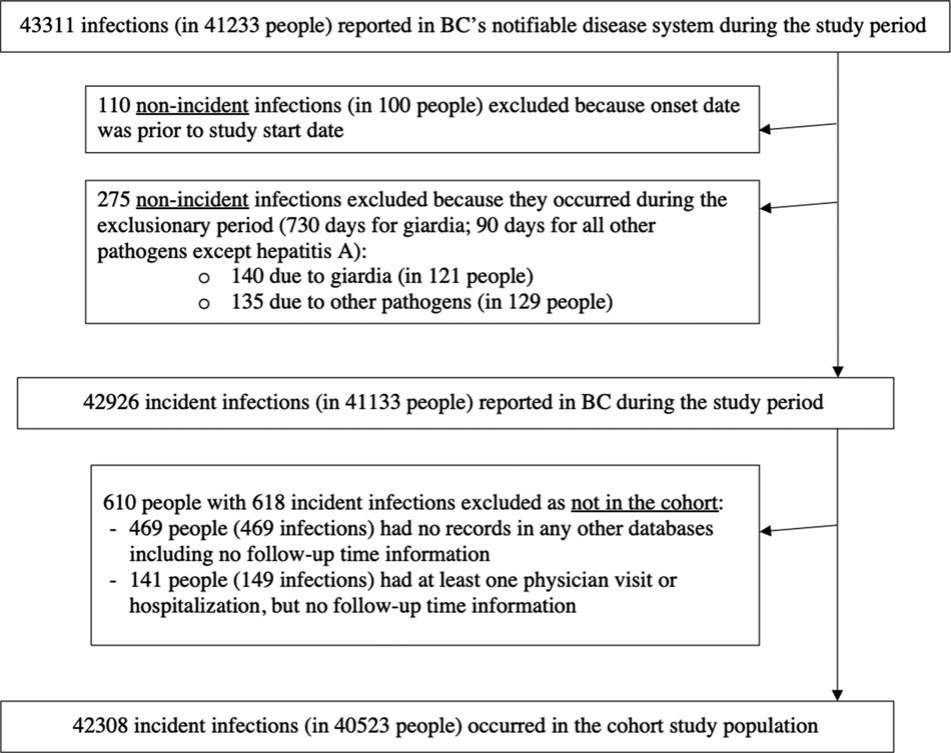
All enteric infections reported in the notifiable disease database in British Columbia (BC), Canada, during the 2005–2014 study period, by incident/prevalent status and ability to link to the population cohort.

From 2005 to 2014 inclusive, the BC public health disease database contained 43 311 reports of infections with the 14 pathogens, in 41 233 individuals. Of these, 110 were excluded as non-incident at the start of the study (Fig. 1).

Visual inspection of campylobacter, non-typhoidal salmonella, shigella and yersinia infection reports revealed that, for individuals with multiple reports for a given pathogen, many of their subsequent reports occurred within 90 days of the first; for giardia, individuals' subsequent infection reports extended over 2 years (Supplementary Table S2). Thus, we set the exclusionary period for campylobacter, non-typhoidal salmonella, shigella and yersinia at 90 days. A 730-day exclusionary period was set for giardia. *C. botulinum* had too few reported infections to allow analysis. For all remaining pathogens, individuals either had zero subsequent reported infections with the same pathogen during the study period (cyclospora, listeria, *Salmonella* Typhi), or these were rare (STEC, *Salmonella* Paratyphi, *Vibrio parahemolyticus*, cryptosporidium; Supplementary Table S2). Thus, for these seven pathogens, and visits and hospitalisations for enteric infections, we chose to use the same 90-day exclusionary period as for campylobacter, etc. Finally, while we explored applying the same exclusionary period to cryptosporidium infections as for giardia, as they are both parasites, all subsequent cryptosporidium reports occurred within 90 days.

Applying these exclusionary periods yielded 275 non-incident reports that were removed from further analysis (Fig. 1, Supplementary Table S3). The remaining 42 926 incident infections reported in BC occurred among 41 133 individuals, of whom only 610 (with 618 incident infections) were not part of our cohort (Fig. 1). These 610 individuals were younger at infection onset, and had a higher proportion of giardia and hepatitis A virus infections, and a lower proportion of campylobacter, non-typhoidal salmonella and yersinia infections compared to the 40 523 individuals in the cohort (Supplementary Tables S3 and S4).

Therefore, our cohort included 5 819 344 individuals who contributed 43 993 114 person-years total follow-up time to our longitudinal study. Individuals were followed for a mean of 7 years and 204 days (standard deviation (s.d.) 3 years and 149 days). The cohort was 50.2% (*n* = 2 918 304) male and had a median age of 33.6 years (IQR 35.5) at their entry into the study. Within the cohort there were 40 523 individuals who had 42 308 incident infections. These individuals contributed a total of 362 944 person-years of follow-up time, with a mean individual follow-up time of 8 years and 349 days (s.d. 2 years and 51 days). We had complete data on age, sex, pathogen and report date for all 42 308 incident infections. As noted above, there were too few reported botulism infections (*n* = 4 during the 10-year study [28–32]) to allow analysis.

**Fig. 2.**
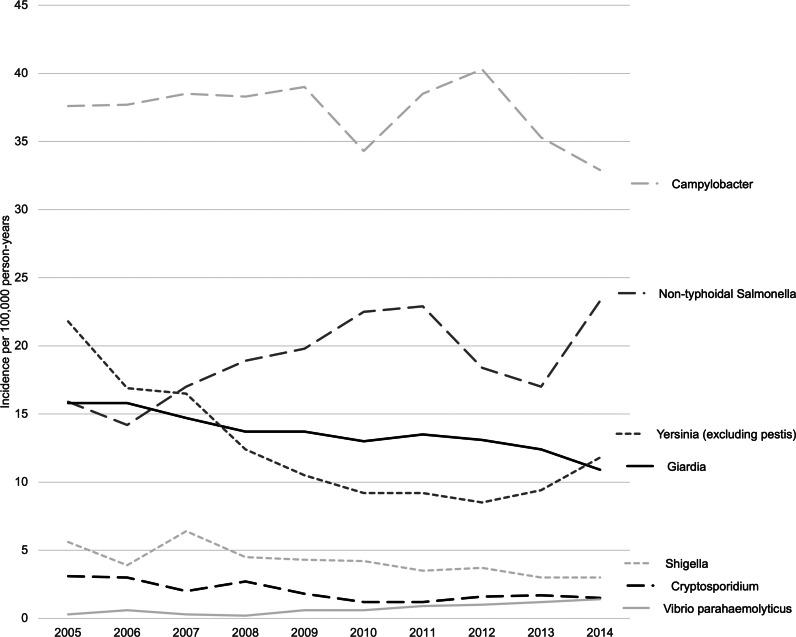
Annual incidence rates of selected enteric infections in a longitudinal cohort in British Columbia, Canada (*n* = 5 819 344), 2005–2014.

There were 96.4 incident infections per 100 000 person-years. The overall incidence of infection was 1.1 times higher for males than females (Table 3). Young children (1–4 years) and young adults (20–24 and 25–29 years) had the highest overall rates, 2.7 and 2.0 times higher than the rates in children aged 10–14 years, respectively. Age-specific differences varied by pathogen, for example, the incidences of cyclospora and *V. parahemolyticus* infections were significantly higher in adults (Table 3). The median age at the onset date of the first incident infection during the study was 37.7 (IQR 33.9) years. Incidence rates and rate ratios, by pathogen, age category and sex are shown (Tables 1 and 3). Campylobacter, non-typhoidal salmonella and giardia had the highest incidence rates, and general trends in rates between 2005 and 2014 varied by pathogen (Fig. 2, Supplementary Table S5). Our pathogen-specific incidence rates agreed with those from provincial surveillance reports (Supplementary Table S6).

**Table 3.** Pathogen-specific incidence rate ratios (95% confidence intervals) for age and sex, in a longitudinal cohort in British Columbia, Canada (*n* = 5 819 344), 2005–2014; significantly higher values are in bold, and significantly lower values in bold italics

Pathogen	Male sex (referent: female)	Age in years									
		<1	1–4	5–9	10–14	15–19	20–24	25–29	30–39	40–59	60+
Total	**1.13** (**1.11–1.16)**	**1.72** (**1.55–1.91)**	**2.73** (**2.57–2.91)**	**1.50** (**1.40–1.60)**	ref.	**1.26** (**1.18–1.34)**	**1.99** (**1.88–2.11)**	**2.04** (**1.92–2.16)**	**1.59** (**1.50–1.68)**	**1.39** (**1.32–1.46)**	**1.37** (**1.30–1.45)**
*Campylobacter*	**1.18** (**1.15–1.22)**	**1.40** (**1.13–1.70)**	**2.61** (**2.33–2.92)**	**1.31** (**1.16–1.48)**	ref.	**1.65** (**1.47–1.84)**	**2.63** (**2.37–2.92)**	**2.55** (**2.30–2.83)**	**1.97** (**1.78–2.17)**	**1.82** (**1.66–2.00)**	**1.96** (**1.78–2.16)**
Non-typhoidal *Salmonella*	1.01 (0.97–1.05)	**3.63** (**3.08–4.27)**	**2.91** (**2.57–3.30)**	**1.59** (**1.39–1.82)**	ref.	**1.22** (**1.07–1.40)**	**1.67** (**1.47–1.89)**	**1.71** (**1.52–1.94)**	**1.14** (**1.01–1.28)**	0.98 (0.88–1.10)	1.04 (0.93–1.16)
*Giardia*	**1.54** (**1.46–1.62)**	0.69 (0.46–1.00)	**3.20** (**2.75–3.73)**	**1.97** (**1.68–2.31)**	ref.	***0.79*** (***0.66–0.95)***	**1.83** (**1.57–2.14)**	**1.93** (**1.66–2.25)**	**1.78** (**1.55–2.06)**	**1.33** (**1.16–1.53)**	***0.74*** (***0.64–0.85)***
*Yersinia* (excl. *pestis*)	***0.82*** (***0.78–0.87)***	**1.71** (**1.21–2.35)**	**2.99** (**2.47–3.63)**	**1.44** (**1.17–1.79)**	ref.	**1.29** (**1.05–1.58)**	**2.09** (**1.74–2.53)**	**2.35** (**1.96–2.83)**	**1.81** (**1.53–2.17)**	**1.91** (**1.63–2.26)**	**2.41** (**2.05–2.86)**
*Shigella*	**1.48** (**1.35–1.62)**	0.85 (0.37–1.69)	**2.38** (**1.71–3.36)**	**1.53** (**1.08–2.19)**	ref.	1.03 (0.72–1.49)	**2.24** (**1.65–3.09)**	**2.68** (**1.98–3.67)**	**2.41** (**1.82–3.25)**	**2.18** (**1.67–2.91)**	1.27 (0.96–1.72)
Shiga toxin-producing *E. coli*	***0.81*** (***0.72–0.90)***	***0.37*** (***0.14–0.77)***	**2.19** (**1.69–2.84)**	1.21 (0.92–1.60)	ref.	1.23 (0.94–1.60)	1.25 (0.96–1.62)	0.77 (0.58–1.03)	***0.54*** (***0.41–0.71)***	***0.49*** (***0.39–0.62)***	***0.76*** (***0.60–0.96)***
*Cryptosporidium*	**1.16** (**1.01–1.33)**	1.24 (0.61–2.28)	**3.82** (**2.79–5.32)**	**2.01** (**1.44–2.85)**	ref.	1.18 (0.82–1.70)	1.19 (0.84–1.72)	**1.61** (**1.16–2.28)**	1.00 (0.73–1.40)	***0.54*** (***0.40–0.75)***	***0.26*** (***0.17–0.38)***
Hepatitis A	1.16 (0.96–1.41)	***0.29*** (***0.04–0.94)***	0.78 (0.46–1.28)	1.11 (0.72–1.70)	ref.	0.74 (0.47–1.15)	0.71 (0.45–1.11)	0.74 (0.47–1.16)	0.70 (0.48–1.03)	***0.39*** (***0.28–0.57)***	***0.25*** (***0.16–0.38)***
*Cyclospora*	1.02 (0.83–1.24)	0.00 (0.00–9.71)	1.86 (0.39–0.11)	1.46 (0.30–7.90)	ref.	**3.40** (**1.07–5.62)**	**6.75** (**2.36–9.41)**	**8.57** (**3.06–7.00)**	**9.22** (**3.44–8.91)**	**8.22** (**3.12–4.42)**	**7.45** (**2.81–1.24)**
*Salmonella* Paratyphi	1.20 (0.98–1.47)	1.02 (0.29–2.66)	**1.84** (**1.07–3.21)**	0.82 (0.43–1.53)	ref.	0.81 (0.44–1.47)	**1.65** (**1.01–2.78)**	1.46 (0.88–2.48)	1.22 (0.77–2.00)	0.72 (0.46–1.16)	***0.42*** (***0.25–0.72)***
*Salmonella* Typhi	1.15 (0.92–1.43)	0.25 (0.01–1.15)	1.69 (1.00–2.86)	0.85 (0.47–1.53)	ref.	***0.34*** (***0.16–0.69)***	1.30 (0.80–2.16)	1.48 (0.92–2.44)	1.00 (0.64–1.61)	***0.48*** (***0.31–0.77)***	***0.19*** (***0.10–0.34)***
*Vibrio parahemolyticus*	**2.24** (**1.78–2.85)**	0.00 (0.00–9.66)	0.00 (0.00–4.91)	0.00 (0.00–3.84)	ref.	2.12 (0.44–6.44)	**3.99** (**1.03–8.42)**	**12.16** (**3.68–81.00)**	**14.10** (**4.45–92.49)**	**10.87** (**3.49–70.40)**	**8.44** (**2.66–54.73)**
*Listeria*	1.27 (0.92–1.77)	**13.55** (**2.79–105.29)**	0.75 (0.02–9.30)	0.00 (0.00–3.84)	ref.	0.88 (0.09–8.49)	0.45 (0.01–5.57)	2.01 (0.41–15.65)	1.38 (0.32–10.20)	2.10 (0.62–14.10)	**11.88** (**3.79–77.16)**

Among individuals with incident infections, 96.0% (38 882/40 523) had one infection during the 10-year study. The remaining 1641 (4.0%) individuals collectively experienced 3426 incident infections. Nearly all (*n* = 1519; 92.6%) experienced two (Table 4), and it was uncommon for individuals to have three (*n* = 105), four (*n* = 12) or five (*n* = 5) incident infections during the 10-year study. Nearly all (1573/1641; 95.9%) of those with multiple incident infections fell into one of three patterns, as follows. Most frequent was having co-infection with more than one pathogen at the same time, once during the study (*n* = 712); these individuals were significantly younger than those with one infection (Table 5). The next most frequent was having more than one infection at different times during the study period, each with a single different pathogen (*n* = 558); significantly more of these individuals were male compared to those with one infection (Table 5). The final pattern was having more than one infection at different times during the study period, all with the same pathogen (*n* = 303); these individuals were significantly older than those with one infection, and significantly more were male (Table 5). For all patterns of multiple infections, the pathogens and combinations occurring most frequently matched the pathogens occurring most frequently in BC (Tables 1, 4–6).

**Table 4.** Pathogen combinations for the 1519 individuals with two incident enteric infections in a longitudinal cohort in British Columbia, Canada (*n* = 5 819 344), 2005–2014; listeria not shown due to cell sizes <5

Pathogen	Total no. individuals (no. individuals with combination reported at the same time^a^)											
	*Campylobacter*	Non-typhoidal *Salmonella*	*Giardia*	*Yersinia* (excluding *pestis*)	*Shigella*	Shiga toxin-producing *E. coli*	*Cryptosporidium*	Hepatitis A	*Cyclospora*	*Salmonella* Paratyphi	*Salmonella* Typhi	*Vibrio parahemolyticus*
*Campylobacter*	116											
Non-typhoidal *Salmonella*	247 (151)	43										
*Giardia*	158 (102)	43 (17)	56									
*Yersinia* (excluding *pestis*)	153 (64)	85 (45)	63 (25)	57								
*Shigella*	99 (74)	20 (14)	77 (39)	21 (10)	19							
Shiga toxin-producing *E. coli*	23 (13)	16 (6)	1–4 (1–4^b^)	14 (7)	6 (1–4)	0						
*Cryptosporidium*	26 (13)	5 (1–4)	32 (22)	7 (1–4)	11 (1–4)	1–4 (1–4)	0					
Hepatitis A	1–4 (1–4)	1–4 (1–4)	1–4 (0)	0	0	0	0	0				
*Cyclospora*	8 (1–4)	1–4 (1–4)	1–4 (1–4)	1–4 (1–4)	1–4 (1–4)	1–4 (1–4)	0	1–4 (0)	0			
*Salmonella* Paratyphi	10 (9)	1–4 (1–4)	7 (6)	1–4 (0)	1–4 (1–4)	0	0	0	0	0		
*Salmonella* Typhi	12 (9)	0	12 (8)	1–4 (1–4)	1–4 (1–4)	0	0	0	0	1–4 (1–4)	0	
*Vibrio parahaemolyticus*	15 (1–4)	1–4 (0)	1–4 (1–4)	1–4 (0)	1–4 (0)	0	0	0	1–4 (0)	0	0	0

a Infections were considered at the same time if onset dates were within 30 days of each other.

b Cell sizes between 1 and 4 presented as 1–4.

**Table 5.** Characteristics of the 40 523 individuals with reported incident enteric infections, by the number of incident infections during the study period, British Columbia, Canada, 2005–2014; *P* values shown compare the group to those with one infection, with significant values bolded

Characteristic	Individuals with 1 infection (*n* = 38 882)	Individuals with >1 infection (*n* = 1641)				
		Everyone with >1 infection (*n* = 1641)	All infections were different pathogens, occurring at the same time^a^ (*n* = 712)	All infections were different pathogens, occurring at different times^b^ (*n* = 558)	All infections were the same pathogen, occurring across the study (*n* = 303)	The remaining *n* = 68^c^
Median age at first onset, in years (IQR); *P* value	37.8 (34.1)	**36.1** (31.1); **0.011**	**30.6** (30.1); **<0.001**	39.5 (30.0); 0.979	**45.1** (29.8); **<0.001**	35.3 (20.9); 0.297
% male (no. individuals); *P* value	51.9 (20 185)	**58.5** (960); **<0.001**	54.4 (388); 0.173	**59.9** (334); **<0.001**	**60.7** (184); **0.002**	**79.4** (54); **<0.001**
Mean no. infections per person (min., max.)	1 (1–1)	2.1 (2–5)	2.0 (2–4)	2.0 (2–4)	2.0 (2–4)	3.3 (3–5)
Mean time between infections, in days (min, max)	–	514 (0–3464)	8.4 (0–30)	898 (31–3337)	979 (94–3464)^d^	579.2 (0–3304)
Most frequent pathogens or pathogen combinations (no. individuals)	–	Campylobacter and non-typhoidal salmonella (264)	Campylobacter and non-typhoidal salmonella (155)	Campylobacter and non-typhoidal salmonella (99)	Campylobacter (122); yersinia (58); giardia (58)	Campylobacter and yersinia (n = 25)
Subset of those with only two incident infections						
No. individuals	–	1519	680–684^e^	545–549	>285	–
Median age at first onset, in years (IQR)	–	37.1 (31.8)	30.6 (29.5)	38.8 (30.2)	43.2 (30.3)	–
% male (no. individuals)	–	57.7 (1752)	54.6 (372)	59.7 (326)	61.0 (178)	–
Most frequent pathogens or pathogen combinations (no. individuals)	–	Campylobacter and non-typhoidal salmonella (247)	Campylobacter and non-typhoidal salmonella (151)	Campylobacter and non-typhoidal salmonella (96)	Campylobacter (116)	–

a Defined as all onset dates within 30 days of each other.

b Defined as all onset dates at least 31 days or more apart from each other.

c 68 individuals had 3 or 4 infections in any other combination of multiple infections.

d Time varied by pathogen, from the shortest, non-typhoidal salmonella (median = 280 days, IQR 229.5), to the longest, giardia (median = 1290 days, IQR 576.3).

e Numbers masked to mask small cell sizes elsewhere in the paper.

**Table 6.** Occurrence of new fee-for-service physician visits and hospitalisations with International Classification of Disease codes for enteric infections (including non-specific infectious gastroenteritis; codes listed in Table 2) among the 244 783 individuals with these events, by whether the individual also had an enteric infection reported in the provincial reportable disease database, British Columbia, Canada, 2005–2014

	Individuals with a reported enteric infection (*n* = 11 263)	Individuals without a reported enteric infection (*n* = 233 520)
No. individuals (%) with the following, with ICD codes for enteric infection:		
Only new physician visits	7591 (67.4)	183 699 (78.7)
Only new hospitalisations	3159 (28.0)	44 421 (19.0)
Both new physician visits and hospitalisations	513 (4.6)	5400 (2.3)
Median no. per person (range):		
Total	1 (1–15)	1 (1–36)
New physician visits	1 (1–15)	1 (1–36)
New hospitalisations	1 (1–8)	1 (1–8)
Median age in years at first visit/hospitalisation (IQR)		
Total	41.0 (36.8)	37.4 (44.9)
New physician visits	37.7 (33.7)	32.9 (43.5)
New hospitalisations	50.5 (42.3)	57.1 (42.3)

Our cohort of 5 819 344 individuals had 544 374 visits and 60 950 hospitalisations with ICD code(s) for enteric infections during the 10-year study period (Table 2). After applying the 90-day exclusionary period, we classified 244 783 individuals (4.2% of the cohort) as having one or more new visits and hospitalisations for enteric infections during the study period (total: 249 945 visits and 56 725 hospitalisations), with a median of 1 per person (range 1–36). Among the 5 778 821 individuals without a reported incident enteric infection during the study, 183 699 (3.2%) had new visit(s), 44 421 (0.8%) had new hospitalisation(s) and 5400 (0.09%) had both new visit(s) and hospitalisation(s) for enteric infections during the 10-year study (Table 6).

Such health care use was more frequent among the 40 523 individuals with reported incident enteric infection(s), where 7591 (18.7%) had new visit(s), 3159 (7.8%) had new hospitalisation(s) and 513 (1.3%) had both, for enteric infections at some point during the 10-year study (Table 6). For these individuals, 81.5% (11 705/14 363) of their new visits and hospitalisations for enteric infections occurred on or after the estimated onset date of the reported infection (Table 7). Half (52.3%, 7509/14 363) occurred on or in the 30 days after infection onset suggesting this was care-seeking related to the laboratory-confirmed, reported infection; extending the window to 90 days post-onset increased the proportion to 56.5% (8113/14 363).

**Table 7.** Number of new fee-for-service physician visits and hospitalisations with International Classification of Disease codes for enteric infections (including non-specific infectious gastroenteritis; codes listed in Table 2), by when the visit/hospitalisation occurred relative to the onset date of the closest laboratory-confirmed, reported enteric infection, among the 11 263 individuals with both types of events, British Columbia, Canada, 2005–2014

	Total no. (*n* = 14 363)		No. physician visits (*n* = 10 422)		No. hospitalisations (*n* = 3941)	
	No.	%	No.	%	No.	%
No. that occurred before the onset date of the reported enteric infection:						
>365 days before infection onset	1378	9.6	1192	11.4	186	4.7
184–365 days before infection onset	282	2.0	236	2.3	46	1.2
91–183 days before infection onset	181	1.3	156	1.5	25	0.6
31–90 days before infection onset	173	1.2	135	1.3	38	1.0
1–30 days before infection onset	644	4.5	435	4.2	209	5.3
No. that occurred on or after the onset date of the reported enteric infection:						
On the same day	163	1.1	96	0.9	67	1.7
1–30 days after infection onset	7346	51.1	4826	46.3	2520	63.9
31–90 days after infection onset	604	4.2	515	4.9	89	2.3
91–183 days after infection onset	408	2.8	325	3.1	83	2.1
184–365 days after infection onset	474	3.3	384	3.7	90	2.3
>365 days after infection onset	2710	18.9	2122	20.4	588	14.9

After applying this 90-day window, our cohort of 5 819 344 individuals included 238 116 individuals with 298 557 new, potentially unreported enteric infections during the study period, including 233 520 individuals with no reported incident infections during the 10-year study, and 4596 individuals with reported enteric infection(s) whose onset dates occurred at a different time (i.e. either after, or more than 90 days before) than their visit/hospitalisation date. Lastly, of the 42 308 reported incident infections in our cohort, 19.2% (*n* = 8113) had a visit or hospitalisation with an ICD code for enteric infection in the 0–90 days following infection onset date.

Our sensitivity analyses revealed that including *vs.* excluding non-incident infections (Supplementary Table S7), and what may have been prevalent infections at the start of the first year of study (2005; Supplementary Table S8) had negligible impacts on our estimated incidence rates.

## Discussion

This study assessed the patterns with which incident enteric infections occurred in a longitudinal population cohort, in the province of BC, Canada between 2005 and 2014. Our cohort included 5.8 million individuals, who were followed for an average of 7.5 years, and captured nearly all enteric infections reported in the province. As expected, the epidemiology closely matches that reported by provincial reportable disease surveillance [26, 28–32]. A small but notable proportion of individuals with reported infections had multiple concurrent infections or more than one infection across the study period. There were also individuals who may have had an enteric infection, though they did not have a concurrent provincially reported laboratory-confirmed infection.

Ninety-nine per cent of the incident infections reported in BC during the study decade were captured in our cohort. Only 610 individuals (with 618 infections) were not registered in the provincial health insurance programme and thus were not in our cohort. These individuals were younger, and had a higher proportion of giardia and hepatitis A virus infections, than those in our cohort. We did not have additional information, but hypothesise they were likely transient in BC (e.g. tourists/visitors, out-of-province students or temporary foreign workers). We also found that our cohort captured sufficient numbers of individuals with infections for 13 of our 14 pathogens of interest, indicating that further assessment (e.g. of sequelae risk) is possible. The exception to this was *C. botulinum*; given there were only four cases during the study period [26, 30–32], we excluded botulism from further assessment.

Most individuals (96%) with incident enteric infections had only one infection during the 10-year study, suggesting that decisions about censoring follow-up time at the date of a second enteric infection may have negligible impact on the comparability of our cohort study to others [8]. If our subsequent sequelae assessments only include an individual's first infection, and exclude anyone with co-infection as others have done [4, 8, 15, 16], this would omit ~720 individuals with co-infection and ignore successive incident infections in ~900 individuals. Unfortunately, other studies to-date (both of sequelae and describing enteric infections overall) do not report the extent of co-infection nor subsequent infections, making comparison challenging. Although omission of these individuals should not impact our ability to detect significant associations between infection and sequelae, we will consider multiple infections when estimating sequelae risk, including whether multiple infections represent a greater risk for sequelae than do one infection with a single pathogen.

Among those who experienced repeated infections with the same pathogen across the study period, the most frequent pathogens (campylobacter, giardia, yersinia and non-typhoidal salmonella) matched the most frequent infections in the province [28–32]. Whether this indicates common or recurrent exposures is unclear and should be explored in future studies. Understanding concurrent infections may also give clues about transmission routes and infection sources. For example, we found co-infections between cryptosporidium and giardia, which may indicate waterborne transmission. Interpreting these findings is challenging given literature is scarce. Outbreaks involving multiple enteric pathogens, while reported, do not appear common [33, 34]. For example, an assessment of outbreaks in the Netherlands reported only five of 5657 outbreaks as having multiple pathogens identified [35]. Additionally, most studies of enteric infections focus on a single pathogen, or use positive reports as their unit of analysis as opposed to individuals (i.e. do not consider reports for different pathogens or reports over time within the same individual).

In our cohort, only 19% of the reported, laboratory-confirmed enteric infections were followed by a visit or hospitalisation for enteric infection in the 0–90 days post-onset. This was unexpected; laboratory testing in BC requires contact with the health care system, and nearly all reported, laboratory-confirmed infections should have had a visit or hospitalisation following onset. However, given that onset dates were missing for 99% of reported infections, necessitating their estimation using median onset-to-reporting times, accurate comparison between infection onset and visit/hospitalisation dates is difficult. As well, we assessed only those visits/hospitalisations with ICD codes for enteric infection, broadly defined, but recognise that such codes are not accurate measures of actual infection [36–38]. Additional explanations may be that our physician visit data include only fee-for-service billings and not visits covered via alternate payment mechanisms (e.g. service contracts [22]), or that our selected ICD codes may not contain codes most commonly used for these visits. Further, 5% of new hospitalisations for enteric infections occurred in the 1–30 days before infection onset, and some proportion would be individuals infected while in hospital. Future studies should assess these issues.

It is likely that some of the 298 557 new physician visits/hospitalisations for enteric infection that occurred without a concurrent reported infection represent incident infections with one of our pathogens of interest. One reason may be that a requested stool sample was not submitted (though what fraction is unknown). Thus, we will include these events as potentially unreported enteric infections, recognising that they may or may not be, and treat this as a separate risk group in future sequelae estimates. To the best of our knowledge, this type of assessment has not yet been done in Canada.

We dealt with the high proportion of missing onset dates in the reportable disease data by estimating infection onset using pathogen-specific median onset-to-reporting times. We will use these estimated onset dates in future sequelae estimates, rather than the dates the infections were reported. Other sequelae studies used stool test date [8, 16], or date of diagnosis or health care use for infection [39, 40] as the start of the time at-risk. Whether our use of an earlier date (i.e. onset of infection, *vs.* the stool testing and health care contact that follows) will generate noticeably longer time-to-sequelae estimates remains unknown.

Finally, we note that our pathogen-specific annual incidence rates did not differ from incidence rates presented in provincial annual surveillance reports, which are calculated as most surveillance reports do, using the number in the population as the denominator. Despite our use of a more exact calculation, differences in rates were negligible, as expected given the low incidence and short duration of illness [25]. Thus, incidence rates calculated in provincial surveillance reports provide excellent estimates of the actual incidence.

In interpreting these results, it is important to note that norovirus and rotavirus, both substantial causes of domestically acquired foodborne and enteric infections in Canada [2], were not captured as incident infections in our study because they are not reportable in BC [26]. Thus, while our study accurately portrays reported infections, the numbers presented here do not capture the full burden of all foodborne and enteric diseases in the province. Norovirus in particular is a leading cause of enteric hospitalisations and deaths in Canada [2, 41], and it is likely responsible for some of the visits and hospitalisations for enteric infection identified here.

Our study has several other limitations. First, we identified incident infections from the provincial reportable disease database, though surveillance systems only capture an estimated one in 10 to one in 100 of the incident infections in the population [2, 23]. This issue is common to registry-based studies of enteric infection sequelae, and we propose to partly overcome this by also assessing individuals who may have had an enteric infection. However, as many enteric infections are mild, with neither reported laboratory confirmation nor physician or hospital visits, we recognise that our findings may not be generalisable to those with mild infections. We chose to use reported infections to ensure we are only including those with confirmed infections in our assessment of sequelae risk. Another potential limitation relates to our choice of exclusionary period, selected based on our data but limited by the fact that the duration of immunity for many pathogens is not well understood [25]. For example, here 303 individuals had multiple infections with the same pathogen across the study period, and it is possible that some of the infections we classified as incident may have been the same infection re-tested more than 90 days later. Finally, the infections in this study were diagnosed by culture and microscopy in most cases. More sensitive diagnostics (e.g. PCR) will increase detections from stool examinations and thus reports of infections [42], which may help reduce non-random misclassification when calculating risk of sequelae.

Despite these limitations, this study confirmed that the incident enteric infections occurring in our cohort accurately capture those reported in the provincial reportable disease database for our target population (the province of BC). As well, our results demonstrate that future sequelae risk analyses should explore the impacts of multiple infections, particularly when more than one pathogen can lead to a sequela. Finally, our study identified a subset of individuals who may have had a potentially unreported enteric infection, and future assessments of the risk of sequelae in this group are warranted.

## Supplementary material

For supplementary material accompanying this paper visit http://doi.org/10.1017/S0950268822001911. click here to view supplementary material
