# Supplementary material for: Patterns of enteric infections in a population-wide cohort study of sequelae, British Columbia, Canada
Source: Epidemiol Infect. 2022 Dec 14;151:e7. doi: 10.1017/S0950268822001911 (PMC9990383; doi:10.1017/S0950268822001911)
Supplement: Supplementary file 1 [file S0950268822001911sup001.docx]

**Epidemiology and Infection**

**ARTICLE: Patterns of enteric infections in a population-wide cohort study of sequelae, British Columbia, Canada**

AUTHORS: Mahmood R. Gohari, Marsha Taylor, Melissa C. MacKinnon, Dimitra Panagiotoglou, Eleni Galanis, Gilaad G. Kaplan, Richard J. Cook, David M. Patrick, Steen Ethelberg, and Shannon E. Majowicz

**Sensitivity Analysis Methods**

We compared pathogen-specific incidence rates in which non-incident enteric infections (either prevalent at the start of the study, or a continuation of an existing infection) were included versus excluded. Next, because a proportion of the incident exposures in the first 89 days of the study (01 January to 30 March 2005; 729 days for giardia) may be a continuation from an exposure at the end of 2004 (i.e., actually non-incident), we examined three different scenarios in calculating the annual incidences for 2005: all incident exposures in the first 89 days of 2005 (729 for giardia) included; all of the same excluded; and a proportion of the same excluded. To estimate the proportion of infections to exclude as potentially prevalent, we estimated the number of infections for each pathogen in the 89 days before our study began (i.e., 04 October to 31 December 2004) by multiplying the average number of infections observed in the last 89 days of the year across our study years (2005-2014) by the observed probability of non-incident exposures for that pathogen during the study (calculated for each pathogen by dividing the number of non-incident exposures by the number of reported infections). Similarly, this was done using 729 days for giardia.

**Supplementary Tables**

**Table S1.** Median number of days between onset date and reported date, for reported enteric infections in British Columbia (BC), Canada, by data source

| **Pathogen** | **Median days between onset date and reported date**  **(no. cases contributing to median / total no. cases)** | | |
| --- | --- | --- | --- |
|  | **Current study**  **(BC’s public health reportable disease database, 2005-2014)** | **Galanis *et al.* (2017) ^a^** | **BC’s public health reportable disease database,**  **2015-2019** |
| *Campylobacter* | 8 (67/16378) | -- | 9 (943/8169) |
| *Clostridium botulinum* | ~^b^ (1-4/1-4) | -- | 8 (5/6) |
| *Cryptosporidium* | 14 (22/858) | -- | 14 (313/487) |
| *Cyclospora* | 20 (21/386) | -- | 19 (182/214) |
| *Giardia* | 23 (16/5995) | -- | 26 (393/2717) |
| Hepatitis A | ~^b^ (1-4/420) | -- | 11 (67/111) |
| *Listeria* | 5 (7/148) | 6 (27) | 5 (48/65) |
| Non-typhoidal *Salmonella* | 10 (238/8385) | 10 (1831) | 10 (3838/5183) |
| *Salmonella* Paratyphi | ~^b^ (1-4/378) | -- | 19 (93/108) |
| *Salmonella* Typhi | ~^b^ (1-4/326) | -- | 11 (133/150) |
| Shiga toxin-producing *E. coli* | 9 (32/1376) | 9 (434) | 11 (529/723) |
| *Shigella* | 10 (59/1843) | 11 (323) | 12 (587/757) |
| *Vibrio parahaemolyticus* | 10 (34/322) | -- | 9 (181/243) |
| *Yersinia* (excluding *pestis*) | ~^b^ (1-4/5490) | -- | 26 (219/3438) |

^a^ Galanis E, *et al*. Evaluating the timeliness of enteric disease surveillance in British Columbia, Canada, 2012-13. Canadian Journal of Infectious Diseases and Medical Microbiology, vol. 2017, Article ID 9854103, 7 pages, 2017. <https://doi.org/10.1155/2017/9854103>

^b^ Unable to calculate median since less than 5 cases reported both onset dates and reported dates

**Table S2.** Time between subsequent report dates (e.g., first to second, or second to third), among individuals who had multiple reports of the same pathogen recorded in the provincial reportable disease database, British Columbia, Canada, 2005-2014

| **Time in days** | **Number (percent) of reports, by pathogen** | | | | |
| --- | --- | --- | --- | --- | --- |
|  | Non-typhoidal *Salmonella*  (n=109) | *Campylobacter*  (n=168) | *Yersinia* (excluding *pestis*)  (n=99) | *Shigella*  (n=50) | *Giardia*  (n=218) |
| 1 to 90 | 59 (54.1) | 25 (14.9) | 31 (31.3) | 12 (24.0) | 19 (8.7) |
| 91 to 180 | 9 (8.3) | 1-10 ^a^ | 1-10 | 1-4 | 16 (7.4) |
| 181 to 270 | 14 (12.8) | 1-10 | 7 (7.1) | 1-4 | 23 (10.6) |
| 271 to 365 | 7 (6.4) | 14 (8.3) | 1-10 | 1-4 | 23 (10.6) |
| 366 to 720 | 12 (11.0) | 50 (29.8) | 23 (23.2) | 15 (30.0) | 61 (28.0) |
| 721+ | 8 (7.3) | 71 (42.3) | 29 (29.3) | 18 (36.0) | 76 (36.2) |

^a^ Numbers masked due to small cell sizes

**Table S3.** The number of enteric infections reported in British Columbia, Canada, 2005-2014, by whether or not the individual was part of our population cohort

| **Pathogen** | **Total no. infections reported (n=43311)** | **No. reported infections excluded because their onset time was in 2004 (n=110)** | **No. reported infections excluded because they were non-incident ^a^ (n=275)** | **No. incident infections excluded because individual could not be linked to the cohort (n=618)** | **Total no. incident infections in the cohort study**  **(n=42308)** |
| --- | --- | --- | --- | --- | --- |
| *Campylobacter* | 16625 | 15 | 24 | 208 | 16378 |
| *Clostridium botulinum* | 4 ^b^ | 0 | 0 | 0 | 4 ^b^ |
| *Cryptosporidium* | 872 | 0 | 1-4 ^c^ | 9-13 | 858 |
| *Cyclospora* | 393 | 0 | 0 | 7 | 386 |
| *Giardia* | 6307 | 42 | 140 | 131 | 5994 |
| Hepatitis A | 441 | 0 | 0 | 21 | 420 |
| *Listeria* | 149 | 0-4 | 0 | 0-4 | 148 |
| Non-typhoidal *Salmonella* | 8540 | 5 | 56 | 94 | 8385 |
| *Salmonella* Paratyphi | 392 | 1-4 | 1-4 | 10 | 378 |
| *Salmonella* Typhi | 337 | 1-4 | 0 | 7-10 | 326 |
| Shiga toxin-producing *E. coli* | 1408 | 0 | 9 | 23 | 1376 |
| *Shigella* | 1897 | 5 | 12 | 37 | 1843 |
| *Vibrio parahaemolyticus* | 331 | 0 | 1-4 | 5-8 | 322 |
| *Yersinia* (excluding *pestis*) | 5615 | 39 | 31 | 55 | 5490 |

^a^ Defined as subsequent infections with the same pathogen with onset dates within 2 years (*Giardia)* or 90 days (all other pathogens)

^b^ There were only 4 reported botulism infections in BC during the 10-year study period, as reported by the British Columbia Centre for Disease Control, Reportable Disease Surveillance Dashboard. Available at: <http://www.bccdc.ca//health-professionals/data-reports/reportable-diseases-data-dashboard>

^c^ Numbers masked due to small cell sizes

**Table S4.** Age and sex of the individuals with incident enteric infections reported in British Columbia, Canada (2005-2014), by whether they were included in our population cohort

| **Characteristic** | **Included individuals**  **(n=40523 with 42308 incident infections)** | **Excluded individuals**  **(n=610 with 618 incident infections) ^a^** |
| --- | --- | --- |
| Median age in years ^b^ (IQR) | 37.7 (33.9) | 26.0 (18.6) |
| Sex n (%) |  |  |
| Male | 21145 (52.2) | 90 (63.8) |
| Female | 19378 (47.8) | 51 (36.2) |

IQR – interquartile range

^a^ Excluded because either: they no records in any of the administrative health databases including no follow-up time information; or they had at least one physician visit or hospitalization, but were not registered in the provincial health insurance program during the study; note that data on sex were missing for most of this group

^b^ Age at the onset date of the first enteric infection reported

^c^ Numbers masked due to small cell sizes

**Table S5.** Annual rates of incident enteric infections in a population cohort in British Columbia, Canada (n=5819344), 2005-2014

| **Pathogen** | **No. incident infections** | **Overall incidence per 100000 person-years ^a^** | **Annual incidence per 100000 person-years** | | | | | | | | | |
| --- | --- | --- | --- | --- | --- | --- | --- | --- | --- | --- | --- | --- |
|  |  |  | **2005** | **2006** | **2007** | **2008** | **2009** | **2010** | **2011** | **2012** | **2013** | **2014** |
| All | 42304 ^b^ | 96.4 | 106.7 | 100.9 | 103.7 | 97.9 | 97.5 | 90.5 | 96.7 | 92.6 | 86.8 | 90.3 |
| *Campylobacter* | 16378 | 37.3 | 37.6 | 37.7 | 38.5 | 38.3 | 39.0 | 34.3 | 38.5 | 40.3 | 35.3 | 32.9 |
| Non-typhoidal *Salmonella* | 8385 | 19.0 | 15.9 | 14.2 | 17.0 | 18.9 | 19.8 | 22.5 | 22.9 | 18.4 | 17.0 | 23.3 |
| *Giardia* | 5994 | 13.7 | 15.8 | 15.8 | 14.7 | 13.7 | 13.7 | 13.0 | 13.5 | 13.1 | 12.4 | 10.9 |
| *Yersinia* (excluding *pestis*) | 5490 | 12.6 | 21.8 | 16.9 | 16.5 | 12.4 | 10.5 | 9.2 | 9.2 | 8.5 | 9.4 | 11.8 |
| *Shigella* | 1843 | 4.2 | 5.6 | 3.9 | 6.4 | 4.5 | 4.3 | 4.2 | 3.5 | 3.7 | 3.0 | 3.0 |
| Shiga toxin-producing *E. coli* | 1376 | 3.1 | 2.7 | 3.6 | 4.3 | 2.7 | 3.5 | 2.4 | 2.4 | 3.2 | 4.0 | 2.5 |
| *Cryptosporidium* | 858 | 2.0 | 3.1 | 3.0 | 2.0 | 2.7 | 1.8 | 1.2 | 1.2 | 1.6 | 1.7 | 1.5 |
| Hepatitis A | 420 | 1.0 | 1.2 | 1.3 | 0.9 | 0.9 | 0.6 | 0.7 | 2.3 | 0.8 | 0.5 | 0.5 |
| *Cyclospora* | 386 | 0.9 | 0.8 | 1.3 | 1.3 | 0.8 | 1.4 | 0.5 | 0.5 | 0.5 | 0.9 | 1.0 |
| *Salmonella* Paratyphi | 378 | 0.9 | 1.1 | 1.5 | 0.8 | 1.1 | 1.1 | 0.7 | 0.8 | 0.7 | 0.5 | 0.5 |
| *Salmonella* Typhi | 326 | 0.7 | 0.6 | 0.9 | 0.7 | 1.2 | 0.9 | 0.7 | 0.7 | 0.7 | 0.5 | 0.6 |
| *Vibrio parahaemolyticus* | 322 | 0.7 | 0.3 | 0.6 | 0.3 | 0.2 | 0.6 | 0.6 | 0.9 | 1.0 | 1.2 | 1.4 |
| *Listeria* | 148 | 0.3 | 0.2 | 0.3 | 0.2 | 0.5 | 0.3 | 0.3 | 0.4 | 0.3 | 0.3 | 0.4 |

^a^ Total of 43993114 person-years of follow-up

^b^ excluding botulism

**Table S6.** Annual incidence of enteric infections in British Columbia (BC), Canada, 2005-2014, (A) calculated from this study (incident infections divided by total person-days at-risk), and (B) reported per BC Centre for Disease Control annual reportable disease surveillance reports (number of reported infections divided by the provincial population)

| **Pathogen** | **Annual incidence per 100000 person-years** | | | | | | | | | | | | | | | | | | | |
| --- | --- | --- | --- | --- | --- | --- | --- | --- | --- | --- | --- | --- | --- | --- | --- | --- | --- | --- | --- | --- |
|  | **2005** | | **2006** | | **2007** | | **2008** | | **2009** | | **2010** | | **2011** | | **2012** | | **2013** | | **2014** | |
|  | **A** | **B ^a^** | **A** | **B ^a^** | **A** | **B ^ab^** | **A** | **B ^a^** | **A** | **B ^a^** | **A** | **B ^a^** | **A** | **B ^ac^** | **A** | **B ^a^** | **A** | **B ^ad^** | **A** | **B ^a^** |
| *Campylobacter* | 37.6 | 37.4 | 37.7 | 37.3 | 38.5 | 38.2 | 38.3 | 37.8 | 39.0 | 39.8 | 34.3 | 34.9 | 38.5 | 38.2 | 40.3 | 40.6 | 35.3 | 35.8 | 32.9 | 33.1 |
| Non-typhoidal *Salmonella* | 15.9 | 15.9 | 14.2 | 14.2 | 17.0 | 17.0 | 18.9 | 18.9 | 19.8 | 19.6 | 22.5 | 22.6 | 22.9 | 23.0 | 18.4 | 19.0 | 17.0 | 17.2 | 23.3 | 23.7 |
| *Giardia* | 15.8 | 16.4 | 15.8 | 15.8 | 14.7 | 15.1 | 13.7 | 14.6 | 13.7 | 13.9 | 13.0 | 14.0 | 13.5 | 13.7 | 13.1 | 13.4 | 12.4 | 13.1 | 10.9 | 11.9 |
| *Yersinia* (excluding *pestis*) | 21.8 | 21.4 | 16.9 | 17.4 | 16.5 | 16.3 | 12.4 | 13.1 | 10.5 | 10.5 | 9.2 | 9.5 | 9.2 | 9.0 | 8.5 | 8.6 | 9.4 | 9.4 | 11.8 | 12.1 |
| *Shigella* | 5.6 | 5.6 | 3.9 | 4.0 | 6.4 | 6.3 | 4.5 | 4.7 | 4.3 | 4.4 | 4.2 | 4.2 | 3.5 | 3.6 | 3.7 | 3.9 | 3.0 | 3.1 | 3.0 | 3.2 |
| Shiga toxin-producing *E. coli* | 2.7 | 2.7 | 3.6 | 3.6 | 4.3 | 4.3 | 2.7 | 2.6 | 3.5 | 3.6 | 2.4 | 2.4 | 2.4 | 2.5 | 3.2 | 3.2 | 4.0 | 4.0 | 2.5 | 2.6 |
| *Cryptosporidium* | 3.1 | 3.0 | 3.0 | 3.0 | 2.0 | 2.1 | 2.7 | 2.6 | 1.8 | 1.9 | 1.2 | 1.2 | 1.2 | 1.2 | 1.6 | 1.6 | 1.7 | 1.6 | 1.5 | 1.6 |
| Hepatitis A | 1.2 | 1.2 | 1.3 | 1.3 | 0.9 | 1.0 | 0.9 | 0.9 | 0.6 | 0.7 | 0.7 | 0.6 | 2.3 | 2.3 | 0.8 | 0.8 | 0.5 | 0.5 | 0.5 | 0.5 |
| *Cyclospora* | 0.8 | 0.8 | 1.3 | 1.2 | 1.3 | 1.4 | 0.8 | 0.7 | 1.4 | 1.4 | 0.5 | 0.6 | 0.5 | 0.5 | 0.5 | 0.5 | 0.9 | 0.8 | 1.0 | 1.0 |
| *Salmonella* Paratyphi | 1.1 | 1.1 | 1.5 | 1.5 | 0.8 | 0.7 | 1.1 | 1.0 | 1.1 | 1.1 | 0.7 | 0.8 | 0.8 | 0.8 | 0.7 | 0.7 | 0.5 | 0.6 | 0.5 | 0.6 |
| *Salmonella* Typhi | 0.6 | 0.6 | 0.9 | 0.9 | 0.7 | 0.7 | 1.2 | 1.2 | 0.9 | 0.9 | 0.7 | 0.7 | 0.7 | 0.7 | 0.7 | 0.7 | 0.5 | 0.5 | 0.6 | 0.7 |
| *Vibrio parahaemolyticus* | 0.3 | 0.4 | 0.6 | 0.7 | 0.3 | 0.3 | 0.2 | 0.5 | 0.6 | 0.8 | 0.6 | 0.9 | 0.9 | 1.0 | 1.0 | 1.2 | 1.2 | 1.4 | 1.4 | 1.5 |
| *Listeria* | 0.2 | 0.2 | 0.3 | 0.3 | 0.2 | 0.2 | 0.5 | 0.5 | 0.3 | 0.3 | 0.3 | 0.3 | 0.4 | 0.4 | 0.3 | 0.3 | 0.3 | 0.3 | 0.4 | 0.4 |

^a^ BCCDC. Reportable diseases data dashboard. Available at: <http://www.bccdc.ca/health-professionals/data-reports/reportable-diseases-data-dashboard> . Accessed 2021-04-15.

^b^ BCCDC. 2007 British Columbia annual summary of reportable diseases. Available at: <http://www.bccdc.ca/resource-gallery/Documents/Statistics%20and%20Research/Statistics%20and%20Reports/Epid/Annual%20Reports/Epid_Stats_Research_CDAnnualReport_2007.pdf> . Accessed 2021-04-13.

^c^ BCCDC. 2011 British Columbia annual summary of reportable diseases. Available at: <http://www.bccdc.ca/resource-gallery/Documents/Statistics%20and%20Research/Statistics%20and%20Reports/Epid/Annual%20Reports/2011_CD_Annual_Report_Final.pdf> . Accessed 2021-04-13.

^d^ BCCDC. 2013 British Columbia annual summary of reportable diseases. Available at: [http://www.bccdc.ca/resource-gallery/Documents/Statistics%20and%20Research/Statistics%20and%20Reports/Epid/Annual%20Reports/2013CDAnnualReportFinal.pdf . Accessed 2021-04-13](http://www.bccdc.ca/resource-gallery/Documents/Statistics%20and%20Research/Statistics%20and%20Reports/Epid/Annual%20Reports/2013CDAnnualReportFinal.pdf%20.%20Accessed%202021-04-13).

**Table S7.** Sensitivity analysis comparing pathogen-specific incidence rates that include or exclude the 275 non-incident exposures in our population cohort, British Columbia, Canada, 2005-2014; listeria and botulism removed due to small cell sizes

| **Pathogen** | **No. incident infections** | **No. non-incident infections ^a^** | **Incidence rate per 100000 person-years** | | |
| --- | --- | --- | --- | --- | --- |
|  |  |  | **Non-incident infections included** | **Non-incident infections excluded** | **Difference in incidence rate** |
| *Campylobacter* | 16378 | 24 | 37.587 | 37.532 | 0.055 |
| *Cryptosporidium* | 858 | 0-4 ^b^ | 1.985 | 1.983 | 0.002 |
| *Cyclospora* | 386 | 0 | 0.888 | 0.888 | 0 |
| *Giardia* | 5994 | 140 | 14.173 | 13.784 | 0.389 |
| Hepatitis A | 420 | 0 | 0.965 | 0.965 | 0 |
| Non-typhoidal *Salmonella* | 8385 | 56 | 19.253 | 19.126 | 0.127 |
| *Salmonella* Paratyphi | 378 | 0-4 | 0.882 | 0.880 | 0.002 |
| *Salmonella* Typhi | 326 | 0 | 0.751 | 0.751 | 0 |
| *Shigella* | 1843 | 12 | 4.283 | 4.255 | 0.028 |
| Shiga toxin-producing *E. coli* | 1376 | 9 | 3.173 | 3.153 | 0.020 |
| *Vibrio parahaemolyticus* | 322 | 0-4 | 0.728 | 0.726 | 0.002 |
| *Yersinia* | 5490 | 31 | 12.849 | 12.775 | 0.074 |

^a^ Defined as subsequent infections with the same pathogen with onset dates within 2 years (*Giardia)* or 90 days (all other pathogens)

^b^ Numbers masked due to small cell sizes

**Table S8.** Sensitivity analysis comparing pathogen-specific incidence rates for 2005, under three scenarios for handling potentially prevalent enteric infections that occurred within the first 90 days (729 for giardia) of the population cohort study, British Columbia, Canada

| **Pathogen** | **No. incident exposures in 2005** | **No. incident exposures within the first 90 days of 2005 (730 days for giardia)**  **(A)** | **Annual incidence rate**  **per 100000 person-years, 2005** | | |
| --- | --- | --- | --- | --- | --- |
|  |  |  | **All of (A) included** | **Proportion ^a^ of (A) excluded** | **All of (A) excluded** |
| *Campylobacter* | 1537 | 295 | 37.386 | 37.375 | 30.739 |
| *Cryptosporidium* | 123 | 21 | 2.962 | 2.962 | 2.529 |
| *Cyclospora* | 34 | 10 | 0.819 | 0.819 | 0.602 |
| Hepatitis A | 48 | 17 | 1.166 | 1.752 | 0.747 |
| *Listeria* | 10 | 1-4 ^b^ | 0.243 | 0.241 | 0.241 |
| Non-typhoidal *Salmonella* | 649 | 158 | 15.750 | 15.745 | 12.115 |
| *Salmonella* Paratyphi | 41 | 13 | 1.060 | 1.059 | 0.723 |
| *Salmonella* Typhi | 23 | 12 | 0.583 | 0.578 | 0.265 |
| *Shigella* | 223 | 53 | 5.535 | 5.417 | 4.335 |
| Shiga toxin-producing *E. coli* | 113 | 16 | 2.721 | 2.717 | 2.408 |
| *Vibrio parahaemolyticus* | 14 | 1-4 ^b^ | 0.340 | 0.337 | 0.313 |
| *Yersinia* (excluding *pestis*) | 847 | 172 | 21.493 | 21.324 | 17.607 |

^a^ See Supplementary Materials – Sensitivity Analysis Methods

^b^ Numbers masked due to small cell sizes
